# Emerging Neurobiological and Therapeutic Insights into Schizophrenia: A Comprehensive Review

**DOI:** 10.3390/ijms27041906

**Published:** 2026-02-16

**Authors:** Anamaria Oatu, Tudor-Florentin Capatina, Iulia-Cristina Mandras, Antonia-Lucia Comsa, Simona Trifu, Arina-Cipriana Pietreanu

**Affiliations:** 1 Department of Psychiatry, Iuliu Hatieganu University of Medicine and Pharmacy, 400012 Cluj-Napoca, Romania; oatu_anamaria@elearn.umfcluj.ro (A.O.); mandras.iulia@gmail.com (I.-C.M.); antonialucia.comsa@yahoo.com (A.-L.C.); 2 Department of Psychiatry, Titu Maiorescu University, 031593 Bucharest, Romania; tudorcapatina98@gmail.com; 3 Department of Neurosciences, Carol Davila University of Medicine and Pharmacy, 020021 Bucharest, Romania; 4 Doctoral School, Carol Davila University of Medicine and Pharmacy, 020021 Bucharest, Romania; arinapietreanu@gmail.com

**Keywords:** schizophrenia neurobiology and pathophysiology, modern treatment guidelines for schizophrenia, precision psychiatry and schizophrenia treatment, negative and cognitive symptoms of schizophrenia, integrated and recovery-oriented schizophrenia care

## Abstract

Schizophrenia is a complex, chronic psychiatric disorder with significant global impact, characterized by persistent positive, negative, and cognitive symptoms that are not fully addressed by current treatments. This review aims to synthesize established theories and advancing mechanistic concepts and also critically compare the latest international treatment guidelines. Recent evidence expands beyond the traditional dopamine hypothesis to include glutamatergic, serotonergic, and cholinergic dysfunctions, as well as emerging mechanisms such as neuroinflammation, oxidative stress, iron dysregulation, and gut–brain interactions. A review of major international guidelines (APA, NICE, CINP, WFSBP, and others) confirms consensus on the use of second-generation antipsychotics as first-line therapy and the early introduction of clozapine for treatment-resistant cases. All guidelines emphasize the essential role of integrated psychosocial interventions, including cognitive behavioral therapy for psychosis, family psychoeducation, and supported employment. Differences remain regarding the prioritization of precision medicine, pharmacogenomics, and digital health innovations. Prognosis varies widely but improves with early intervention, sustained treatment adherence, and comprehensive physical health monitoring. Overall, schizophrenia care is evolving toward a precision-based, recovery-oriented model that integrates biological, psychological, and social strategies to improve long-term outcomes and quality of life.

## 1. Introduction

Schizophrenia is a severe, chronic psychotic disorder that typically emerges in late adolescence or early adulthood and is associated with substantial disability, reduced life expectancy, and high health-care and societal costs [1,2]. Global estimates derived from the Global Burden of Disease (GBD) programme indicate that schizophrenia affects tens of mil-lions of people worldwide; although age-standardized rates are relatively stable across regions, the absolute burden has increased over time due to population growth and ageing [2].

Clinically, schizophrenia is best conceptualized as a syndrome spanning three partially dissociable symptom domains: positive symptoms (e.g., delusions and hallucinations), negative symptoms (e.g., avolition, anhedonia, asociality, blunted affect, and alogia), and cognitive impairment (including deficits in processing speed, attention, working memory, learning and memory, and executive function) [3,4]. Negative and cognitive symptoms are major determinants of real-world functioning and quality of life and often persist even when positive symptoms improve with antipsychotic treatment [3,4].

Over the past decades, theoretical models have evolved from a dopamine-centric account toward multidimensional, circuit-based frameworks. Contemporary neurotransmitter models integrate dopaminergic dysregulation with glutamatergic and GABAergic disturbances and downstream network-level alterations, aligning with dysconnectivity hypotheses and neurodevelopmental vulnerability–stress formulations [5,6]. In parallel, data-driven approaches emphasize biological heterogeneity: biomarker-based stratification studies suggest that clinically similar psychosis presentations can arise from partially distinct neurobiological profiles, motivating efforts to subtype illness beyond di-agnostic categories [7,8].

Despite major advances, several gaps in the last decade of schizophrenia research continue to limit translation to improved outcomes. First, treatment development has outpaced mechanistic understanding for many non-positive symptom dimensions: negative symptoms remain an unmet therapeutic target, and cognitive deficits are a core feature that accounts for much long-term disability yet shows limited responsiveness to current antipsychotics [3,4,9]. Second, schizophrenia is highly heterogeneous, but clinically actionable biomarkers for prognosis and treatment selection remain limited, and findings across omics, neuroimaging, and digital phenotyping often do not generalize beyond specific cohorts [8]. Third, although immune and inflammatory mechanisms have been implicated in subgroups of patients, clinical trials of adjunctive anti-inflammatory strategies show mixed results, reinforcing the need for stage-specific, stratified trials with replication in larger, diverse samples [10].

Accordingly, this review synthesizes established theories alongside advancing mechanistic concepts (including neuro-transmitter, circuit, immune-inflammatory, and gut–brain frameworks) and critically compares major international treatment guidelines. By highlighting convergent evidence and unresolved controversies, we aim to clarify the biological and clinical rationale for current standards of care and to outline priorities for next-generation, mechanism-based and recovery-oriented interventions.

## 2. Results

### 2.1. Current Theories of Schizophrenia

#### 2.1.1. The Dopamine Hypothesis

**Figure 1:**
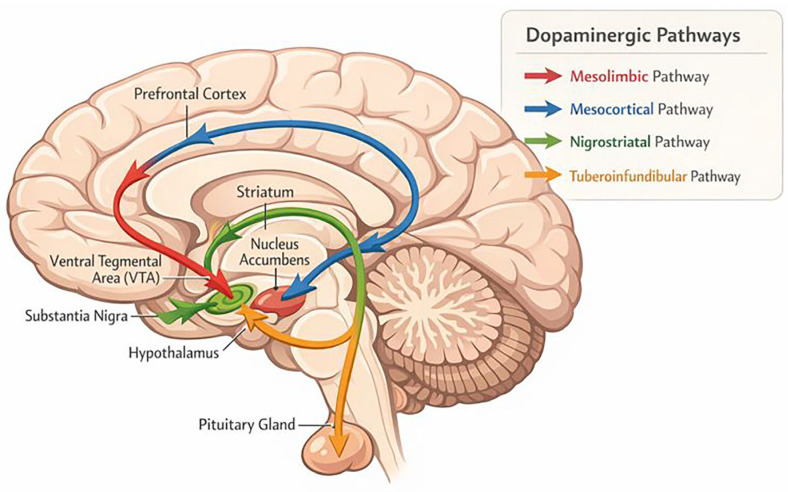
Dopamine pathways in the brain.

One of the first neurochemical models of schizophrenia emerged from observations that dopamine receptor antagonists, particularly those targeting D2 receptors, have antipsychotic properties [1]. There are 5 main pathways that are affected by the dopamine malfunction in this theory (Figure 1):

1. The nigrostriatal dopamine pathway that projects from substantia nigra to the basal ganglia or striatum which is part of the extrapyramidal nervous system and controls motor functions and movement.
2. The mesolimbic pathway arises from the ventral tegmental area (VTA) of the midbrain and projects to limbic regions, including the nucleus accumbens. It plays a central role in the brain’s reward circuitry and mediates processes such as motivation, pleasure, and reinforcement. Hyperactivity of this pathway is thought to contribute to the positive symptoms of schizophrenia, including delusions and hallucinations [11,12,13,14].

3. The mesocortical dopaminergic pathway originates in the ventral tegmental area (VTA) of the midbrain and projects to the prefrontal cortex, where it modulates both cognition and affective processes. Within this circuit, the dorsolateral prefrontal cortex (dlPFC) is principally involved in executive and cognitive functions, including working memory and decision-making, whereas the ventromedial prefrontal cortex (vmPFC) is implicated in emotional and affective processing. Hypoactivity or reduced dopaminergic signaling in these mesocortical regions, particularly within the dlPFC, has been associated with the negative and cognitive symptoms observed in schizophrenia [6,14,15,16].
4. Tuberoinfundibular dopaminergic pathway connects the hypothalamus to the anterior pituitary gland and plays a key role in the tonic inhibition of prolactin secretion via dopamine acting on D2 receptors. Pharmacological blockade of D2 receptors in this pathway, as occurs with many antipsychotic medications, disrupts this inhibitory control and can result in hyperprolactinemia. In contrast, basal dopamine secretion is generally considered to be within the normal range in untreated schizophrenia, with dopaminergic abnormalities primarily reflecting dysregulated presynaptic dopamine synthesis and release rather than elevated baseline dopamine levels [6,17,18]
5. The fifth one arises from multiple places, including the periaductal gray, ventral mesencephalon, hypothalamic nuclei, and lateral parabrachial nucleus, and projects to the thalamus. Although its precise function remains unclear, evidence suggests it may be involved in regulating arousal and sleep by modulating thalamocortical information processing [19].

Although studies indicate increased dopamine synthesis in prodromal phase and the first episode of schizophrenia, there is typically a delayed therapeutic response of 2–4 weeks between the peak D2 receptor blockade and the onset of clinical improvement. This has led to the development of alternative hypotheses of schizophrenia [1].

Recent molecular imaging meta-analyses have refined classical dopaminergic models of schizophrenia by demonstrating that presynaptic dopamine dysfunction is not confined to the mesolimbic system, but is often most pronounced in associative and dorsal striatal subdivisions. These findings challenge a strict mesolimbic-centric framework and suggest a more distributed and functionally heterogeneous dopaminergic abnormality underlying psychotic symptoms [6,20].

#### 2.1.2. The GABAergic Hypothesis

**Figure 2:**
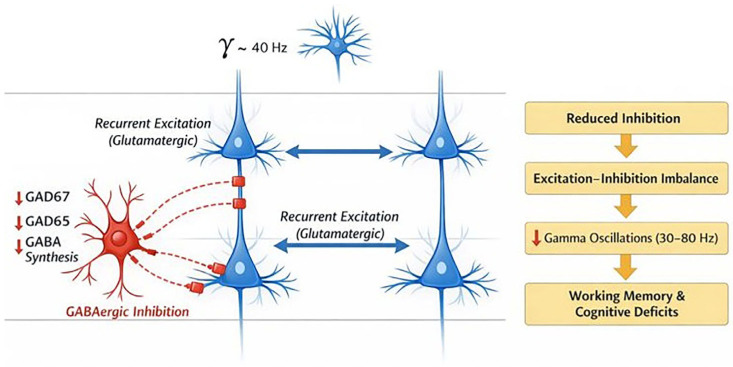
GABAergic interneuron dysfunction and excitation–inhibition imbalance in prefrontal cortical microcircuits implicated in schizophrenia.

Another hypothesis involves dysregulation of the GABA neurotransmitter system. Accumulating evidence points to altered GABAergic transmission in the prefrontal cortex (PFC) of individuals with schizophrenia, a disturbance that may contribute to impairments in cognitive functions such as working memory [21]. Post-mortem studies have further demonstrated that, within the PFC, GABAergic interneurons expressing the calcium-binding protein parvalbumin exhibit reduced mRNA expression per cell [22]. In addition, decreased levels of mRNA and protein for the 67 kDa isoform of glutamate decarboxylase (GAD67), an enzyme critical for GABA synthesis, have been shown to be diagnostically specific for schizophrenia [23]. Beyond GAD67, reduced expression of GAD65 has also been observed in GABAergic terminals [24]. As illustrated schematically in Figure 2, these molecular alterations in parvalbumin-positive interneurons are thought to weaken perisomatic inhibitory control over pyramidal neurons, contributing to excitation–inhibition imbalance within prefrontal cortical microcircuits and potentially disrupting cortical network dynamics relevant to cognitive function.

To better understand this hypothesis, experiments in mice have shown that reduced GABA synthesis alone does not cause schizophrenia [25]. In this sense, studies indicate that reducing the actin-cytoskeleton regulatory protein Arp2/3 in the prefrontal cortex leads to a progressive loss of dendritic spines, followed by molecular and behavioral alterations resembling those seen in schizophrenia, highlighting how structural and synaptic abnormalities in the PFC may interact with inhibitory dysfunctions to contribute to the disorder [26].

**Figure 3:**
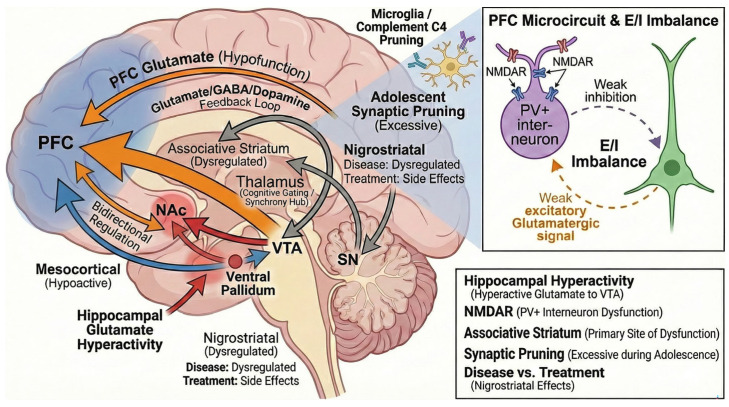
Integrated neurodevelopmental and circuit model of schizophrenia pathophysiology.

To better understand the role and implications of GAD67 described above, an additional study has investigated its involvement in mouse models. The study showed that suppression of GAD67 leads to elevated corticosterone levels and lower birth weight compared to wild-type littermates. Postnatal analyses showed a reduced number of PV-expressing neurons in the PFC of GAD67 heterozygous knockout mice exposed to maternal stress. In contrast, these mice exhibited decreased sociability and aggressiveness even in the absence of maternal stress. Also, social isolation testing indicated increased locomotor activity in GAD67 heterozygous knockout mice, an effect not observed in their wild-type counterparts (Figure 3) [27]. Figure 3 illustrates a multilevel model integrating neurodevelopmental, cellular, and circuit-based mechanisms underlying schizophrenia and related psychotic disorders. Hypofunction of glutamatergic signaling in the prefrontal cortex (PFC), potentially driven by NMDA receptor (NMDAR) abnormalities, contributes to impaired top-down regulation of subcortical dopaminergic systems. Reduced cortical excitatory output disrupts cortico-striatal-thalamo-cortical (CSTC) feedback loops, resulting in dysregulated dopamine signaling and impaired cognitive control. NMDAR hypofunction on parvalbumin-positive (PV+) GABAergic interneurons reduces inhibitory control of pyramidal neurons within the PFC microcircuit. This produces excitation–inhibition (E/I) imbalance, impaired cortical synchrony, and disrupted network oscillatory activity, contributing to cognitive and negative symptom domains. Hyperactive glutamatergic output from the hippocampus enhances striatal dopamine neuron activity through a polysynaptic pathway involving the nucleus accumbens (NAc) and ventral pallidum (VP). Increased hippocampal drive disinhibits ventral tegmental area (VTA) dopaminergic neurons, promoting dopaminergic hyperactivity in striatal circuits. The associative striatum is highlighted as the principal region demonstrating dopamine dysregulation in psychosis, consistent with human molecular imaging studies. Altered dopaminergic signaling within this region contributes to aberrant salience attribution and positive symptom expression. The thalamus functions as a cognitive gating and synchrony hub within CSTC loops. Dysconnectivity between thalamic nuclei and cortical regions disrupts information filtering, attentional regulation, and cognitive integration. Mesocortical dopamine projections to the PFC are relatively hypoactive and associated with executive dysfunction and negative symptoms. Mesolimbic dopaminergic projections to ventral striatal structures exhibit relative hyperactivity, contributing to psychotic symptomatology. These pathways are regulated by reciprocal cortical–subcortical feedback mechanisms. The nigrostriatal pathway is depicted as dysregulated in disease states and is additionally susceptible to pharmacological dopamine receptor blockade, which may produce extrapyramidal motor side effects during antipsychotic treatment. Excessive synaptic pruning during adolescence, potentially mediated by microglial activation and complement cascade signaling (including complement component C4), contributes to synaptic loss and cortical circuit disruption. These developmental processes may interact with genetic vulnerability and environmental risk factors to promote illness onset.

Another important factor is immune activation induced by psychological stress, which can, in turn, influence the progression of the disease. Although the presence of GABA-A receptors in microglia remains unclear, microglia express GABA-B receptors that can modulate K+ permeability in response to GABA signaling [28]. Microglia release interleukin-6 (IL-6) in response to lipopolysaccharide (LPS) stimulation; however, this release is inhibited by activation of GABA-B receptors [29]. Loss of microglia-specific GABA-B receptors has been shown to significantly impair GABAergic synaptic pruning. Furthermore, as the animals reach adulthood, a marked reduction in GABAergic terminals suggests a potential association with schizophrenia [30].

### 2.2. Advancing Concepts and Mechanisms

#### 2.2.1. Serotonin Dysregulation in Schizophrenia

The initial link between serotonin (5-HT) and schizophrenia emerged from the structural similarity between 5-HT and the hallucinogenic compound lysergic acid diethylamide (LSD). Further support for the involvement of serotonergic mechanisms came with the introduction of clozapine, an atypical antipsychotic shown to be more effective than typical antipsychotics [31]. Its superior clinical efficacy has been attributed to its antagonistic activity at 5-HT_2_ receptors, as well as to the combined antagonism of 5-HT_2_ and dopamine D_2_ receptors [32].

5-HT is a neurotransmitter involved in numerous behavioral and physiological processes, many of which are disrupted in schizophrenia. These include cognition (such as memory, perception, and attention), sensory gating, mood regulation, aggression, sexual behavior, appetite, energy balance, pain sensitivity, endocrine function, and sleep [33]. Disturbances in several of these domains correspond to core features of schizophrenia and contribute to both its positive and negative symptomatology.

Alterations in 5-HT transmission in schizophrenia is supported by post-mortem studies. Most affected were the 5-HT transporters density and an increase in 5-HT1A receptor binding [34]. Also, a decrease in 5-HT2A density has been frequently observed, but it may be due to neuroleptic exposure [35,36,37]. Recent work on the molecular underpinnings of schizophrenia highlights the interplay between neuroinflammation and neurotransmitter systems—including serotonin (5-HT) pathways—suggesting that inflammatory processes may modulate serotonergic signaling in psychotic disorders and could inform future therapeutic strategies for schizophrenia [38].

Recently, it was observed that modification of the endogenous agonist serotonin can markedly alter the Gαq protein coupling profile of the 5-HT_2_A receptor (5-HT_2_AR) and the corresponding behavioral outcomes. Notably, among these outcomes, we show that memory deficits are specifically modulated by Gα1 (subunit of the G protein) [39].

#### 2.2.2. The Acetylcholine Hypothesis

Evidence for disturbances in cholinergic neurotransmission, involving both muscarinic and nicotinic receptors, is reflected in the increased prevalence of smoking among individuals with schizophrenia. This phenomenon has led to the hypothesis that patients may use nicotine as a form of self-medication to compensate for underlying neurochemical imbalances [40,41]. Treatment with nicotine or other nicotinic cholinergic agonists appears to normalize certain eye-tracking and electroencephalographic (EEG) abnormalities observed in individuals with schizophrenia and may also enhance specific aspects of cognitive function [42].

Similarly to smoking, some studies have observed that individuals with schizophrenia who chew betel nut exhibit fewer positive symptoms compared to non-chewers [43]. To assess the extent of involvement of these receptors, a postmortem study has further demonstrated reduced muscarinic M_1_ and M_4_ receptor binding in the cortical, hippocampal, and striatal regions of individuals with schizophrenia compared to healthy controls. Moreover, this reduction appears to follow a distinct pattern from that observed in other disorders, such as Alzheimer’s disease and major depressive disorder [44].

Individuals with schizophrenia also show greatly decreased upregulation of high affinity nicotinic acetylcholine receptors (nAChRs) as a result of smoking compared to control subjects, indicating that the high prevalence of smoking in this population may be driven by a reduced nicotinic effect on this receptor subtype. This is also backed by the fact that all antipsychotic medication can reduce nAchR binding [45,46].

Dysregulated muscarinic acetylcholine receptor (mAChR) signaling was associated with schizophrenia, with postmortem studies showing decreased receptor expression in the prefrontal cortex, hippocampus, and other brain regions [47]. Xanomeline, an agonist at muscarinic M_1_ and M_4_ receptors administered in combination with the peripheral anticholinergic trospium to improve tolerability, significantly reduces scores on the Positive and Negative Syndrome Scale (PANSS) compared with placebo in individuals with schizophrenia [48].

Despite these findings, nicotinic acetylcholine receptors influence multiple neurotransmitter systems, and it remains unclear whether cholinergic dysfunction in schizophrenia is a primary disturbance or secondary to other pathological features. In addition, muscarinic antagonists can induce psychosis-like symptoms in healthy individuals and exacerbate existing symptoms in patients with schizophrenia, highlighting the critical role of muscarinic signaling in the disorder [49,50].

### 2.3. Current Treatments and Prognosis

Schizophrenia is a chronic and multifactorial psychiatric disorder characterized by positive, negative, and cognitive symptoms, affecting approximately 1% of the global population. The disorder significantly impairs social, occupational, and functional outcomes, making it one of the leading causes of disability worldwide [51,52]. Although the neurobiological mechanisms of schizophrenia remain incompletely understood, converging evidence implicates dopaminergic dysregulation, glutamatergic dysfunction, neuroinflammatory processes, and genetic vulnerability [53]. Modern treatment guidelines increasingly emphasize the importance of integrating biological, psychological, and social perspectives into a recovery-oriented model that aims not only for symptom remission but also for improved functioning and quality of life [54,55,56].

Over the past decade, major international bodies have updated their treatment recommendations to reflect advancements in pharmacotherapy, digital health, and psychosocial interventions. These include the American Psychiatric Association (APA, the National Institute for Health and Care Excellence (NICE), the World Federation of Societies of Biological Psychiatry (WFSBP), the Collegium Internationale Neuro-Psychopharmacologicum (CINP), and the McCutcheon European update. Despite substantial overlap among these guidelines, significant differences persist regarding antipsychotic selection, the management of treatment-resistant schizophrenia (TRS), and the integration of non-pharmacologic modalities [57,58,59,60,61]. Treatment-resistant schizophrenia (TRS) is now operationally defined by consensus criteria requiring inadequate response to at least two antipsychotic trials of adequate dose, duration, and adherence, as established by the Treatment Response and Resistance in Psychosis (TRRIP) working group. This definition has become the standard reference in both clinical trials and guideline development [62]. The current review aims to synthesize and critically compare these major recommendations, emphasizing both the established evidence base and the emerging gaps that guide future research.

#### 2.3.1. Pharmacological Treatment in Schizophrenia

- Treatment objectives and clinical principles

Antipsychotic pharmacotherapy is the core evidence-based treatment for schizophrenia, reducing symptom severity and—when continued as maintenance—substantially lowering relapse risk. Maintenance treatment is also associated with benefits in patient-centered outcomes such as functioning and quality of life, supporting its role in long-term management [63,64]. The 2024 APA guideline emphasizes a personalized, measurement-based approach that integrates pharmacologic, psychosocial, and recovery-oriented strategies [63].

According to Stahl (2021), effective treatment relies on aligning neurobiological targets with the clinical symptom profile, a principle referred to as mechanism-based prescribing [19]. This involves understanding receptor affinities (dopamine, serotonin, histamine, adrenergic, muscarinic) and their clinical correlates (e.g., sedation, weight gain, akathisia, prolactin elevation).

The standard algorithm across guidelines includes:

1. Initiation: A single antipsychotic trial (4–6 weeks at therapeutic dose);
2. Optimization: Dose adjustment and adherence verification (weeks 6–12);
3. Switching: Transition to a different antipsychotic if response < 20–25%;
4. Treatment Resistance: Initiation of clozapine after ≥2 failed trials [34,35,36,37,38].

- Pharmacological classes and mechanisms

First-generation antipsychotics (typical antipsychotics) exert their antipsychotic effects primarily through potent dopamine D_2_ receptor antagonism, which effectively reduces positive symptoms of schizophrenia; however, this mechanism also underlies a greater propensity for extrapyramidal side effects and a higher risk of tardive dyskinesia compared with later agents [65,66]. Common first-generation antipsychotics (typical antipsychotics) include haloperidol, fluphenazine, chlorpromazine, perphenazine, and zuclopenthixol, all of which have been used in the treatment of schizophrenia and related psychotic disorders [67,68,69]. Dose range: haloperidol (2–10 mg/day), fluphenazine (5–20 mg/day).

While APA and NICE do not exclude their use, they are typically reserved for patients with good past response or cost constraints. Stahl (2021) highlights that strong D_2_ blockade (>80%) produces EPS and secondary negative symptoms by diminishing striatal dopaminergic tone [19].

Second-generation antipsychotics generally achieve antipsychotic efficacy at moderate D_2_ receptor occupancy (~60–70%) and, through prominent 5-HT_2_A antagonism, tend to show improved neurological tolerability compared with stronger D_2_-predominant blockade; cognitive improvements, where present, are typically modest. They form the first-line pharmacologic recommendation in all major guidelines [63,70,71]. For a comparative overview of the clinical profiles and adverse effects of SGAs, see Table 1.

Clinical practice guidelines commonly recommend maintaining antipsychotic treatment at the lowest effective dose to sustain response and reduce adverse effects, including cardiometabolic risk. Typical effective adult dose ranges used in schizophrenia studies include risperidone 2–6 mg/day, olanzapine 10–20 mg/day, aripiprazole 10–30 mg/day, and lurasidone 37–148 mg/day [80,81,82,83]. Guidelines suggest annual metabolic and neurologic screening; APA integrates digital tools for longitudinal monitoring [63].

- Novel and emerging mechanisms

Partial D_2_ Agonists: drugs such as Aripiprazole, Brexpiprazole, and Cariprazine act as dopamine “stabilizers.” Stahl (2021) emphasizes their ability to preserve physiological dopaminergic signaling, reducing both EPS and anhedonia [19]. Cariprazine: D_3_ preference, beneficial for negative and cognitive symptoms; CINP (2023) recommends it in early psychosis or residual symptoms [84]. Brexpiprazole: has a better tolerability, improved mood regulation.

New-generation non-D_2_ drugs, such as ulotaront (TAAR1/5-HT_1_A agonist), represent a paradigm shift. They offer efficacy in both positive and negative symptoms without EPS or metabolic risk [85]. Xanomeline–trospium, a muscarinic M_1_/M_4_ agonist combination, is another promising compound targeting cortical circuits. These agents appear in McCutcheon (2025) as next-wave therapies potentially redefining schizophrenia pharmacotherapy [61].

- Clozapine and treatment resistance

Clozapine remains the gold standard for the management of treatment-resistant schizophrenia [86,87], with strong consensus across clinical guidelines supporting its use after two adequate trials of non-clozapine antipsychotics, each lasting at least six weeks [86]. Initiation requires careful titration, typically beginning at 12.5 mg and gradually increasing to a therapeutic range of approximately 300–600 mg per day, corresponding to plasma levels of 350–600 ng/mL [19,88,89]. Owing to its safety profile, clozapine treatment mandates rigorous monitoring, including weekly absolute neutrophil count assessments for the first six months, followed by biweekly or monthly monitoring thereafter [87]. Clinicians must remain vigilant for serious adverse effects such as agranulocytosis, myocarditis, severe constipation, and metabolic disturbances [19,86]. For patients with suboptimal response, augmentation strategies may be employed, including the addition of aripiprazole or amisulpride to target persistent negative symptoms [90], or mood stabilizers such as valproate for affective instability [86]. Electroconvulsive therapy may be considered in ultra-resistant cases [91,92,93]. Importantly, early initiation of clozapine—ideally within the first three years of illness—has been associated with improved long-term outcomes, including reduced hospitalization rates and lower suicide-related behaviors compared with later initiation or use of other antipsychotics [94,95]. In other words, longitudinal cohort studies published in the last decade indicate that delayed initiation of clozapine in individuals with TRS is associated with poorer long-term outcomes, including significantly higher rates of psychiatric rehospitalization. Conversely, earlier clozapine initiation appears to confer sustained clinical stability, supporting recommendations for more timely recognition of treatment resistance and earlier clozapine use [94].

Beyond its efficacy in treatment-resistant illness, clozapine remains unique among antipsychotic agents in its association with reduced suicide risk and lower all-cause mortality. Large population-based studies and meta-analyses consistently demonstrate that clozapine-treated patients exhibit lower rates of suicide attempts and deaths compared with those receiving other antipsychotics or no antipsychotic treatment.

- Long-Acting Injectable (LAI) Antipsychotics

LAIs are recommended early when adherence is questionable. Options: paliperidone palmitate (monthly or 3-month), aripiprazole lauroxil, olanzapine pamoate [96,97,98]. Key benefits: 30–50% relapse reduction, consistent plasma levels, and decreased rehospitalization [99,100,101].

McCutcheon (2025) stresses that LAIs should be offered via shared decision-making to avoid coercion [61,86].

- Polypharmacy and augmentation

Although antipsychotic polypharmacy is generally discouraged by guidelines, treatment-resistant schizophrenia affects roughly 20–30% of patients, and carefully selected combination strategies are sometimes used when adequate monotherapy (including clozapine) is insufficient. Cohort evidence and large comparative analyses most consistently support clozapine augmented with aripiprazole for relapse-related outcomes, while randomized evidence supports adding low-dose aripiprazole to risperidone to reduce antipsychotic-induced hyperprolactinemia; adjunct aripiprazole combinations have also been associated with improvements in metabolic parameters in some controlled data [102,103,104]. Adjunctive antidepressants are frequently used in schizophrenia when comorbid depressive symptoms are clinically prominent; meta-analyses of add-on antidepressants (including SSRIs among commonly studied/used agents) have evaluated benefits and safety, and large real-world data confirm antidepressants are widely co-prescribed in schizophrenia care [105,106,107]. Mood stabilizers (most commonly valproate) are sometimes used adjunctively in schizophrenia, particularly when clinicians are targeting behavioral dyscontrol such as aggression/hostility or affective instability; randomized and real-world evidence suggests valproate augmentation may reduce aggression measures in some patients [108,109]. For patients who remain refractory, non-pharmacological augmentation strategies such as ECT or rTMS have shown benefit in some studies—particularly for persistent symptoms such as clozapine-resistant psychosis or auditory verbal hallucinations—although results are mixed across trials and meta-analyses, underscoring the importance of individualized, multimodal treatment planning [110,111,112,113,114].

- Pharmacogenomics and personalized prescribing

Polymorphisms in CYP2D6 and CYP1A2 can alter psychotropic drug exposure and are associated with adverse-effect risk, while DRD2 and HTR2A variants have been linked to variability in antipsychotic/antidepressant response and specific side-effect domains [19,115,116,117]. Evidence supports the clinical utility of pharmacogenomic testing where available—particularly for metabolism-related CYP450 genes—to optimize dosing of psychiatric medications. The 2023 Clinical Pharmacogenetics Implementation Consortium (CPIC) guideline provides dosing recommendations based on CYP2D6 and CYP2C19 genotypes for antidepressants. Clinical primers and implementation studies further emphasize how CYP450 genotype information can guide choice and dose in real-world psychiatric practice [118,119,120]. Stahl advocates for therapeutic drug monitoring (TDM) combined with genetic data to guide titration and minimize non-response [19].

Management of schizophrenia requires tailored approaches for specific populations to optimize safety and efficacy. In first-episode psychosis, lower doses of antipsychotics are often effective and associated with better tolerability, and expert panels note the importance of early conservative dosing in FEP. Second-generation antipsychotics (SGAs) are typically preferred as initial agents due to lower risk of extrapyramidal side effects than first-generation drugs, and agents such as lurasidone demonstrate particularly favorable metabolic profiles [121,122]. In elderly patients with schizophrenia, minimizing anticholinergic burden and metabolic risk is a clinical priority, as high anticholinergic load is associated with cognitive impairment and adverse outcomes in this population and high antipsychotic doses correlate with increased mortality. Agents with lower metabolic liability are preferred; in contrast, clozapine and olanzapine are associated with marked metabolic side effects and should generally be avoided in older adults unless clearly indicated [123,124]. In pregnancy, antipsychotic treatment should aim for the lowest effective dose to balance maternal mental health and fetal risk, as pregnancy alters drug pharmacokinetics and the evidence base indicates that this approach optimizes outcomes. Typical antipsychotics such as haloperidol are among the most studied and are often considered relatively safe for use when antipsychotic therapy is necessary, and olanzapine has not been consistently associated with increased major congenital malformation risk in observational studies [125,126,127]. In individuals with comorbid substance use disorders (SUDs), antipsychotic selection often balances psychotic symptom control with safety and tolerability. Partial dopamine agonists such as aripiprazole and cariprazine have demonstrated good control of psychotic symptoms as well as favorable safety profiles in schizophrenia with comorbid SUDs, and preliminary clinical data suggest potential reductions in substance use severity alongside symptom improvement. Their unique pharmacodynamics, including partial agonism at D2/D3 receptors, may underlie better tolerability and lower risk of misuse compared with some full antagonists [128,129].

Effective management of antipsychotic adverse effects is essential to optimize treatment adherence and long-term outcomes. Metabolic complications should be proactively addressed through regular monitoring of body mass index, waist circumference, fasting glucose, and lipid profiles, with metformin considered to mitigate antipsychotic-induced weight gain when appropriate [56]. Extrapyramidal symptoms are best managed with short-term use of anticholinergic agents such as benztropine, avoiding prolonged exposure whenever possible [130]. In cases of hyperprolactinemia, switching to a partial dopamine agonist can help normalize prolactin levels while maintaining antipsychotic efficacy [131]. Sedation may be minimized by adjusting dosing schedules to the evening or transitioning to a more activating antipsychotic [132]. For medications associated with QT interval prolongation, particularly ziprasidone and haloperidol, regular electrocardiographic monitoring is recommended to reduce the risk of cardiac complications [133].

Maintenance antipsychotic treatment is central to long-term schizophrenia management and reduces relapse risk. In first-episode/remitted patients, multiple guidelines and reviews commonly recommend continuing antipsychotic treatment for at least 1–2 years (often operationalized as ~2 years) after remission, whereas for individuals with recurrent episodes or chronic illness, guidelines frequently support long-term—often effectively indefinite—maintenance treatment [64,134]. Any consideration of dose reduction should occur only after a minimum of 12 months of sustained clinical stability and must be undertaken gradually under close clinical supervision. The American Psychiatric Association (2024) strongly cautions against abrupt discontinuation, as evidence indicates that more than half of patients experience relapse within one year when treatment is stopped suddenly [63].

#### 2.3.2. Non-Pharmacological and Psychosocial Interventions

While pharmacological treatments remain central to schizophrenia management, psychosocial and behavioral interventions are essential for promoting long-term recovery, social reintegration, and reduction in relapse risk. All major guidelines emphasize their implementation as a core component of comprehensive care.

Cognitive-Behavioral Therapy for Psychosis (CBTp) is recommended across all reviewed guidelines as an evidence-based adjunct to pharmacotherapy. The APA (2020) advocates CBTp for patients with persistent positive symptoms despite antipsychotic use, focusing on restructuring maladaptive beliefs and improving coping strategies [135]. NICE (2023) specifies that CBTp should be offered to all individuals with psychosis, ideally involving at least 16 planned sessions, with a focus on normalizing psychotic experiences, enhancing insight, and reducing distress [55]. The CINP highlights CBTp’s role in addressing cognitive biases and meta-cognitive deficits, supporting integration with digital delivery methods for improved accessibility [136]. Cognitive Behavioural Therapy for psychosis (CBTp) and related CBT-derived approaches (e.g., metacognitive training) target maladaptive cognitions and cognitive biases that contribute to psychotic symptoms. Meta-analytic evidence shows positive impacts on cognitive biases and symptom severity in schizophrenia, and these interventions are conceptualized to address distorted thinking processes. Digital formats such as web-based and videoconference CBTp have been studied, supporting feasibility and accessibility improvements for delivering CBTp in broader clinical settings [137]. Meanwhile, the WFSBP (2019) recommends CBTp primarily for residual symptoms and relapse prevention, whereas McCutcheon et al. (2025) emphasize AI-assisted, hybrid CBT models, which incorporate symptom tracking and virtual feedback to tailor therapy intensity [61].

Family psychoeducation is a key factor in relapse prevention and functional recovery. According to NICE (2023), family interventions should consist of at least 10 sessions, involving all household members when possible [55]. The APA (2024) supports structured family therapy focused on communication skills, stress reduction, and relapse signs, while WFSBP (2019) stresses the importance of engaging families early in treatment to mitigate stigma and caregiver burden [56]. Recent updates, including McCutcheon et al. (2025), propose integrating digital psychoeducation platforms and peer-support networks, aligning with the modern shift toward collaborative, recovery-based care [61].

Cognitive deficits in schizophrenia, particularly in attention, memory, and executive functioning, predict long-term disability. Cognitive remediation therapy (CRT) aims to enhance neurocognitive performance through structured exercises and compensatory strategies. The CINP (2023) and APA (2024) recommend CRT as part of early intervention programs, especially when paired with supported employment and social skills training [63,136]. NICE (2023) highlights behavioral social skills training (SST) as an effective adjunct, focusing on communication, problem-solving, and adaptive functioning [55]. The McCutcheon (2025) model advances CRT by including digital gamified platforms and AI-based monitoring to improve engagement and personalization [61].

Functional recovery remains incomplete without occupational reintegration. Individual Placement and Support (IPS) programs are endorsed across guidelines as the most effective model for achieving employment outcomes [138]. APA (2024) and NICE (2023) highlight IPS as an integral component of recovery-oriented care, supported by multidisciplinary coordination among psychiatrists, psychologists, and social workers. WFSBP (2019) recommends stepwise reintegration for patients with severe cognitive or negative symptoms, while CINP (2023) supports the use of digital employment coaching tools for remote follow-up [56,136].

A growing body of research supports digital mental health platforms, mindfulness-based interventions, and physical activity programs as valuable adjuncts. NICE (2023) explicitly recommends structured exercise and arts therapies to improve negative symptoms and quality of life [55]. APA (2024) encourages telepsychiatry and smartphone-based symptom tracking, while McCutcheon (2025) explores the potential of digital phenotyping, using behavioral and sensor data to monitor relapse risk and personalize interventions [61]. The CINP (2023) further suggests blended approaches integrating mobile cognitive training, relaxation modules, and AI-driven monitoring, aligning with the evolution toward precision psychosocial care [136].

#### 2.3.3. Integrated and Recovery-Oriented Care Models

Traditional models of schizophrenia care have focused primarily on symptom remission. However, recovery-oriented care expands this focus to include personal empowerment, autonomy, and social participation. The APA (2024) defines recovery as “a process through which individuals improve their health and wellness, live a self-directed life, and strive to reach their full potential” [63]. NICE (2023) and McCutcheon (2025) both stress co-production of care, where patients are active agents in treatment planning, contributing to higher satisfaction and adherence [87,136].

All guidelines acknowledge the necessity of multidisciplinary, coordinated care models. The WFSBP (2019) promotes integrated networks connecting psychiatry, primary care, and community services [56]. CINP (2023) and APA (2024) highlight the value of early intervention services, especially in the first episode of psychosis, combining pharmacologic, psychological, and social strategies under unified care teams [63,136]. McCutcheon (2025) advances this further by proposing AI-assisted integrated care models, which merge clinical data, digital biomarkers, and self-report outcomes to optimize treatment sequencing and predict relapse [61].

A comparative synthesis of recommendations across major schizophrenia guidelines is presented in Table 2.

**Figure 4:**
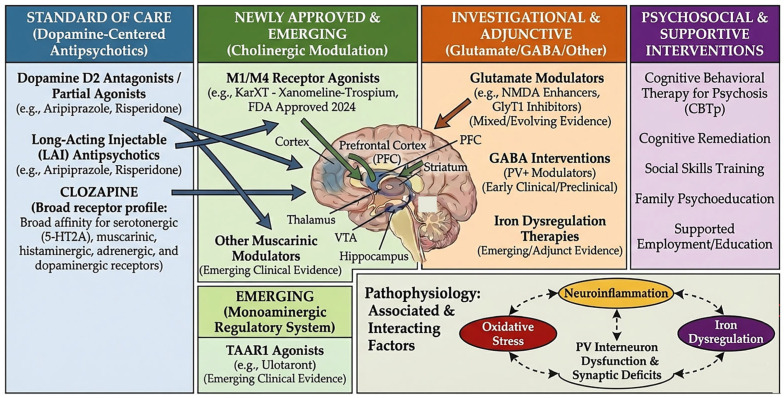
Contemporary therapeutic landscape for schizophrenia.

Schematically in Figure 4, contemporary models of schizophrenia treatment emphasize the integration of multiple neurobiological targets with both pharmacological and psychosocial interventions within a recovery-oriented framework. Dysregulation across dopaminergic, GABAergic, glutamatergic, serotonergic, and cholinergic systems provides the biological rationale for current and emerging pharmacological strategies, including second-generation antipsychotics, partial dopamine agonists, clozapine, and novel non-D_2_ agents [53,60,61,62,63,64,65,66]. In parallel, evidence-based psychosocial interventions—such as cognitive-behavioral therapy for psychosis, cognitive remediation therapy, family psychoeducation, and supported employment—address cognitive, functional, and social dimensions of the disorder and are essential complements to pharmacotherapy [46,47,53,115]. Rather than implying linear or deterministic relationships, Figure 4 illustrates how converging biological and psychosocial interventions are associated with improvements across positive, negative, and cognitive symptom domains, ultimately supporting functional recovery and quality of life. Current schizophrenia treatment strategies are organized by pharmacologic mechanism and clinical implementation stage. Dopamine-centered antipsychotics, including D2 receptor antagonists and partial agonists, remain first-line treatments for psychosis. Long-acting injectable formulations improve adherence and reduce relapse risk. Clozapine is the gold-standard therapy for treatment-resistant schizophrenia and exerts effects through broad multireceptor activity involving serotonergic, muscarinic, histaminergic, adrenergic, and dopaminergic systems. Novel non-dopaminergic approaches include muscarinic receptor agonists targeting M_1_/M_4_ signaling and trace amine-associated receptor 1 (TAAR1) agonists that modulate monoaminergic neurotransmission through dopamine-independent mechanisms. Investigational adjunctive therapies target glutamatergic and GABAergic circuit dysfunction, including NMDA receptor modulation, glycine transporter inhibition, and parvalbumin interneuron–focused strategies, although clinical evidence remains evolving. Additional emerging approaches explore the role of iron dysregulation as a contributor to oxidative stress and neuroinflammatory processes. Psychosocial interventions—including cognitive behavioral therapy for psychosis, cognitive remediation, social skills training, family psychoeducation, and supported employment and education—remain essential components of comprehensive treatment. Figure 4 also highlights interacting biological processes implicated in schizophrenia pathophysiology, including neuroinflammation, oxidative stress, iron dysregulation, and inhibitory interneuron dysfunction, which collectively contribute to synaptic and circuit-level abnormalities.

#### 2.3.4. Future Directions in Current Guidelines

A major direction for future schizophrenia research lies in precision psychiatry, which aims to individualize treatment based on biological, digital, and psychosocial markers. The McCutcheon et al. (2025) report emphasizes that heterogeneous treatment response across patients cannot be fully explained by dopamine dysregulation alone [61]. Instead, integrating neuroimaging data, genomic risk scores, and inflammatory biomarkers could refine therapeutic matching and predict both efficacy and adverse outcomes.

The CINP (2023) and APA (2024) guidelines recommend advancing biomarker-guided treatment algorithms, while also warning against premature implementation without replication in large cohorts [63,136]. Emerging evidence implicates NRG1/ErbB signaling in clozapine-treated and treatment-resistant schizophrenia, including prospective biomarker work suggesting NRG-1 may track or predict clozapine response. Genetic studies of clozapine response remain inconsistent overall (including candidates such as BDNF), and epigenetic markers (e.g., NR3C1 methylation and broader methylation signatures) are being investigated as potential components of future multi-omic stratification approaches [139,140]. Moreover, digital phenotyping, which refers to the use of smartphone and wearable data to infer mental states, has gained traction as a non-invasive complement to biological measures [141]. When integrated with clinical monitoring, these tools could enable continuous, adaptive treatment optimization.

Despite progress in managing positive symptoms, negative and cognitive symptoms remain refractory to most current treatments. The WFSBP (2019) identifies this as a “therapeutic blind spot”, urging the development of new pharmacological and psychosocial tools [56]. Recent trials with TAAR1 agonists (ulotaront) and glutamatergic agents show preliminary efficacy in reducing avolition and anhedonia, but reproducibility remains limited [85]. Non-pharmacologic options such as cognitive remediation therapy (CRT), social cognition training, and aerobic exercise have demonstrated modest benefits [55,136]. Future work must explore synergistic interventions that combine neuromodulation, digital training, and psychotherapy within an integrated framework.

AI (Artificial Intelligence) is emerging as a pivotal tool for advancing schizophrenia care. McCutcheon et al. (2025) propose using machine learning algorithms to predict relapse, personalize dosing, and flag early warning signs from digital health data [61]. APA (2024) and CINP (2023) acknowledge that AI tools can enhance clinical decision support but stress the ethical need for transparency, interpretability, and patient autonomy [63,136]. The introduction of AI-driven CBT platforms, digital monitoring systems, and virtual reality-based rehabilitation heralds a shift toward tech-enabled recovery ecosystems, where human clinicians and digital interfaces collaborate dynamically.

Implementation of evidence-based schizophrenia care varies widely across countries, reflecting differences in health-system infrastructure, workforce capacity, and financing. Community-based models (including ACT/ICM and integrated care approaches) are more established in many high-income settings, whereas many low- and middle-income regions face persistent barriers including limited access/affordability of psychotropic medicines, stigma-related barriers to engagement, and shortages of trained mental health professionals [142,143,144]. NICE (2023) emphasizes system-level reform and task-shifting, empowering non-specialist workers to deliver psychosocial care [55]. The WFSBP (2019) calls for culturally adaptable interventions and scalable technologies, ensuring equity in access to both pharmacologic and psychosocial modalities [56].

Recovery in schizophrenia is evolving from a symptom-based construct to a multidimensional process encompassing clinical, functional, and personal domains.

APA (2024) defines recovery as individualized and dynamic, while McCutcheon et al. (2025) argue for outcome metrics that capture subjective well-being, social participation, and digital engagement [61,63]. The next generation of guidelines will likely incorporate patient-reported outcome measures (PROMs) and digital experience sampling as essential endpoints, bridging the gap between clinical efficacy and lived experience.

#### 2.3.5. Schizophrenia Prognosis

Schizophrenia is a chronic but heterogeneous psychiatric disorder whose course and outcome vary widely among individuals. Modern clinical guidelines converge on the view that prognosis is multifactorial, influenced by biological vulnerability, duration of untreated psychosis, treatment adherence, and social context rather than by diagnosis alone [55,87].

All major guidelines highlight that schizophrenia’s prognosis is highly variable. A significant subset of patients achieves substantial recovery, particularly with early and sustained intervention [55,87]. NICE (2023) estimates that around one-third of individuals with first-episode psychosis attain symptomatic remission within the first year, while another third follow a fluctuating course and the remaining third experience chronic impairment [55]. The APA (2024) guideline similarly stresses that functional recovery, defined as sustained employment, independent living, and social participation, lags behind symptomatic control and must be targeted explicitly [63]. McCutcheon et al. (2025) further emphasize that heterogeneity of response underscores the need for stratified and personalized treatment algorithms that integrate biological and psychosocial variables [61].

Consistent prognostic factors appear across the CINP, NICE, and WFSBP documents.

Favorable predictors include: shorter duration of untreated psychosis (DUP), good premorbid adjustment (academic and social), acute onset of symptoms, strong family or community support, and adherence to evidence-based pharmacological and psychosocial treatments [55,56,136].

Poor prognostic indicators include: long DUP, insidious onset, predominant negative or cognitive symptoms, comorbid substance misuse, persistent treatment resistance, and social adversity [56,63,136]. Early intervention services and assertive outreach are therefore central recommendations in all recent guidelines to mitigate these risks and promote recovery [55,63,87].

Relapse prevention remains one of the most critical determinants of long-term prognosis. The WFSBP and APA guidelines recommend continuous antipsychotic maintenance, at the lowest effective dose and for an individualized duration, combined with psychosocial interventions such as CBTp, family psychoeducation, and supported employment [56,63]. Meta-analytic data summarized by CINP (2023) show that maintenance therapy reduces relapse rates by approximately 60%, particularly when adherence is supported through long-acting injectable antipsychotics [136]. For TRS, clozapine remains the gold-standard therapy with superior efficacy for symptom reduction, relapse prevention, and suicide risk mitigation when initiated promptly [56,63]. Delays in clozapine initiation are consistently linked to poorer long-term outcomes [87].

While symptom remission is achievable in many cases, functional recovery, the ability to sustain relationships, work, and independent living, remains the major unmet challenge.

Negative symptoms and cognitive impairment emerge from the reviewed literature as primary determinants of long-term functional disability in schizophrenia. Despite their clinical importance, these symptom domains remain poorly responsive to existing pharmacological treatments, representing a major unmet therapeutic need. Recent reviews emphasize that progress in this area has been limited by symptom heterogeneity, measurement challenges, and insufficient mechanistic targeting [4,145].

NICE (2023) and CINP (2023) stress that pharmacological treatment alone is insufficient; recovery-oriented programs combining individual placement and support (IPS), cognitive remediation, and social skills training significantly improve functional outcomes [55,136]. The APA (2024) also underscores the importance of integrated multidisciplinary care, including case management, physical health monitoring, and vocational rehabilitation [63]. McCutcheon et al. (2025) highlight that integrated early intervention teams producing individualized recovery plans yield measurable gains in both functional and symptomatic dimensions [61].

All guideline bodies express strong concern about the markedly reduced life expectancy in schizophrenia, estimated at 10–20 years shorter than the general population, primarily due to cardiometabolic disorders, smoking, sedentary behavior, and inequitable access to medical care [56,146]. APA schizophrenia guidance emphasizes routine monitoring of cardiometabolic risk factors (e.g., weight/BMI, blood pressure, and metabolic laboratory parameters) during antipsychotic treatment, and contemporary guidance also supports structured lifestyle interventions (diet/physical activity/behaviour change) as part of care to reduce metabolic and cardiovascular risk in people with schizophrenia/SMI [146]. Physical-health optimization is framed not only as a medical imperative but also as a determinant of psychiatric prognosis and recovery [146].

McCutcheon et al. (2025) identify future priorities for improving prognosis: precision psychiatry approaches (pharmacogenomics, imaging biomarkers) to predict treatment response; digital relapse prediction tools using mobile monitoring; and large-scale health-system integration to ensure equitable delivery of early intervention [61]. However, all guidelines caution that these innovations remain adjuncts, not substitutes, for comprehensive, person-centered care that addresses both biological and social determinants.

Across all authoritative sources, the consensus is that schizophrenia prognosis is variable but modifiable. Timely access to coordinated care, sustained antipsychotic therapy, early use of clozapine for TRS, and integration of psychosocial and physical-health interventions are the key modifiable factors. Long-term outcomes depend as much on systemic factors such as service accessibility and social inclusion as on individual biology. Thus, prognosis improves most reliably when treatment systems embody a recovery-oriented, multidisciplinary, and continuous-care model, as endorsed by APA, NICE, CINP, WFSBP, and McCutcheon et al. [56,61,63,87,136,146].

Taken together, these findings indicate that the convergence of pharmacologic, psychosocial, and recovery-oriented paradigms marks a critical evolution in schizophrenia care. Across the reviewed guidelines, several foundational pillars consistently emerge: early, sustained pharmacologic treatment with individualized antipsychotic selection; integration of psychosocial interventions, particularly CBTp, family psychoeducation, and supported employment; multidisciplinary, recovery-oriented frameworks that promote patient agency and community reintegration.

Key divergences lie in antipsychotic hierarchy, digital integration, and recovery metrics. While APA and CINP emphasize clinical pharmacology and precision medicine, NICE foregrounds accessibility and co-production of care. McCutcheon et al. introduce an innovative synthesis of AI, digital phenotyping, and biomarker-based prediction, while WFSBP remains grounded in traditional hierarchical pharmacotherapy.

Future progress depends on addressing unresolved challenges: optimizing treatment-resistant cases, mitigating negative and cognitive symptoms, and ensuring global accessibility to integrated, recovery-oriented services.

As psychiatry enters the era of precision and digital mental health, the synthesis of these guidelines provides a unified foundation for evidence-based, person-centered schizophrenia treatment in the 21st century.

### 2.4. Future Directions in Treatment and Research

The future of psychosis research and treatment is defined by a shift toward precision, personalization, and prevention. Advances in neuroscience, immunology, and digital health are shaping a new paradigm that moves beyond symptom management to address underlying mechanisms and long-term outcomes.

The classical dopaminergic hypothesis of psychosis is being supplemented by emerging models that emphasize neuroinflammation, oxidative stress, and immune system dysregulation. Future research is focusing on identifying biological subtypes of psychosis using genetic, inflammatory [147,148], and neuroimaging biomarkers [149]. Although immune and inflammatory mechanisms have been implicated in schizophrenia pathophysiology, randomized controlled trials of anti-inflammatory adjunctive treatments have yielded mixed results. Recent meta-analyses suggest that therapeutic benefit may be restricted to biologically defined subgroups or specific illness stages, underscoring the need for biomarker-driven stratification in future trials [10,150].

Stratifying patients based on these biological dimensions may lead to more targeted interventions. For example, individuals with elevated inflammatory markers may benefit from adjunctive anti-inflammatory therapies, such as minocycline or N-acetylcysteine [151,152]. Similarly, pharmacogenomic profiling could inform the choice and dosing of antipsychotic medication, minimizing side effects and improving treatment response [153].

Recent findings also support the existence of at least two biological subtypes of schizophrenia, referred to as type A and type B. Type A is characterized by increased striatal dopamine activity and a good response to antipsychotic medications that block dopamine receptors, whereas type B shows normal dopamine synthesis and a poor response to such treatments [154]. However, growing evidence indicates that this two-type classification may not fully capture the disorder’s complexity. Future research should aim to identify additional subtypes beyond the A/B model, potentially linked to glutamatergic dysregulation, oxidative stress, or immune system mechanisms.

Beyond the dopaminergic A/B framework, recent multimodal neuroimaging work using quantitative susceptibility mapping and [18F]-DOPA PET has shown that reduced iron levels in the substantia nigra–ventral tegmental area are inversely correlated with striatal dopamine synthesis, suggesting that iron deficiency may represent a distinct biological pathway contributing to dopaminergic dysregulation [155]. Complementary QSM and diffusion imaging studies further indicate that patients with schizophrenia show reduced subcortical iron and myelin levels, particularly within the basal ganglia and SN–VTA, implicating oligodendrocyte dysfunction [156]. Moreover, dysregulated iron homeostasis may exacerbate oxidative stress and trigger ferroptotic mechanisms, potentially contributing to the neurobiological pathology of schizophrenia [157]. Together, these findings highlight an emerging iron–myelin–dopamine axis that may represent a novel biological dimension of the disorder, offering new targets for mechanism-based interventions aimed at restoring cellular metabolism and neural connectivity.

Recent bioinformatic analyses of peripheral blood from first-episode, drug-naïve schizophrenia patients identified three immune-related predictive genes (FOSB, NUP43, and H3C1) associated with neutrophil and resting natural killer cell proportions [158]. Consistent with prior evidence of altered natural killer cell activation and impaired cytotoxic function in first-episode psychosis, these transcriptomic signatures further support immune dysregulation as a key feature of the disorder [159]. Collectively, the integration of RNA sequencing and machine-learning approaches demonstrates strong potential for identifying molecular biomarkers and stratifying patients into biologically informed subtypes.

Environmental factors further contribute to mechanistic heterogeneity. Elevated exposure to toxic heavy metals, particularly lead (Pb) [160], has been associated with increased schizophrenia risk, potentially via inflammatory pathways involving TNF, IL-1β, and altered TP53 expression. This evidence underscores the interplay between environmental and molecular factors in shaping biologically distinct subgroups [161].

Further, machine-learning techniques applied to peripheral inflammatory biomarkers have been used to classify patients into treatment-response subgroups: antipsychotic-responsive, clozapine-responsive, and clozapine-resistant. These approaches reveal subtle biological signals and heterogeneity that traditional statistics may overlook, demonstrating the potential of biomarker-driven predictive models to guide more targeted and effective interventions [162].

Emerging adjunctive interventions targeting novel biological mechanisms, such as probiotics, may modulate the gut–brain axis and reduce clinical symptoms, offering additional pathways for personalized treatment strategies beyond traditional dopamine-based therapies [163].

For over half a century, the dopamine hypothesis has served as the dominant framework for understanding and treating schizophrenia. While dopaminergic antagonists remain the cornerstone of pharmacotherapy, their limited efficacy for negative and cognitive symptoms underscore the need for novel mechanistic perspectives [164].

Recent advances in molecular psychiatry and neuroimaging have expanded our understanding of schizophrenia as a disorder involving distributed neural circuit dysfunctions that extend beyond dopamine dysregulation [165]. Emerging evidence implicates glutamatergic signaling, GABAergic balance, neuroinflammatory processes, and mitochondrial integrity as key contributors to disease pathophysiology [166,167,168,169]. These discoveries are catalyzing a shift toward mechanism-based and precision-guided approaches, where imaging and molecular biomarkers are integrated to refine patient stratification and identify new therapeutic targets [170].

Interpretation of neuroimaging biomarkers in schizophrenia must carefully account for the confounding effects of antipsychotic exposure. Recent placebo-controlled crossover studies in healthy volunteers demonstrate that even short-term administration of dopaminergic agents such as amisulpride and aripiprazole can transiently increase striatal volume, with changes reversing after drug discontinuation. These findings indicate that at least part of the volumetric alterations observed in medicated patients may reflect pharmacological rather than disease-related effects [171]. Notably, recent longitudinal work in first-episode, drug-naïve patients further revealed that both pre- and post-treatment striatal volumes, particularly within the left nucleus accumbens, predict cognitive improvement following antipsychotic therapy, underscoring region-specific and treatment-dependent effects on brain morphology [172]. This underscores the critical need to differentiate medication-induced neuroanatomical changes from those intrinsic to the disorder when interpreting imaging data. Future research should thus disentangle illness-specific neurobiological signatures from treatment-induced changes to accurately define biological subtypes and mechanistic pathways.

At the same time, functional neuroimaging is providing valuable insights into treatment response and potential therapeutic targets. Arterial spin labeling (ASL) MRI studies in treatment-resistant schizophrenia have shown reduced frontal and parietal cerebral blood flow (CBF) prior to clozapine initiation, with baseline striatal and hippocampal perfusion predicting the degree of symptomatic improvement following treatment. Moreover, longitudinal decreases in anterior cingulate and thalamic CBF have been associated with poorer clinical outcomes, indicating that alterations in cerebral perfusion may serve as dynamic biomarkers of treatment responsiveness [173]. Together, this body of evidence reinforces the broader shift toward mechanism-based and precision-guided therapeutics in psychosis.

Advances in large-scale neuroimaging consortia, particularly through the ENIGMA collaboration, have established reproducible patterns of cortical thinning and subcortical volume abnormalities in schizophrenia. While these findings have strengthened circuit-based models of disease, their clinical utility remains limited by substantial interindividual variability [174].

fMRI studies reveal structural and functional disruptions in key brain regions, including the prefrontal cortex, hippocampus, and default mode network (DMN), which correlate with cognitive and emotional deficits. AI techniques, including machine learning, deep learning, and explainable AI approaches, enhance the detection and analysis of these complex neural patterns [175]. Explainable AI can uncover individualized brain patterns associated with schizophrenia, sex differences, and brain aging, providing deeper insights into neurobiological mechanisms and informing precision diagnostics and tailored interventions [176].

Beyond neuroimaging, AI-powered digital tools support clinical management in multiple ways. Machine-learning (ML) algorithms applied to smartphone-based adherence monitoring can predict medication-taking behavior, helping clinicians optimize treatment and reduce relapse risk [177]. Similarly, generative AI and ML systems have shown promise in automating the assessment of negative symptoms, including expression and motivation/pleasure domains, offering objective, efficient, and reliable alternatives to traditional clinical evaluations [178].

Recent studies have also explored rhythmic digital markers (RDMs), capturing behavioral rhythms across ultradian, circadian, and infradian timescales, as potential digital phenotypes for schizophrenia. Dynamic RDMs derived from transitions in activity and symptom intensity can differentiate patients from controls, highlighting the promise of digital behavioral monitoring to complement traditional assessments and support precision psychiatry [179].

Recent studies highlight key determinants of recovery in schizophrenia, including individual factors (e.g., speech, employment hope, introspective accuracy), novel interventions such as MERIT and MERITg, and the role of a strong therapeutic alliance [180]. High-risk states, social engagement, empathy, and metacognition also appear to influence recovery, while multimodal approaches (e.g., EEG) may help elucidate the neural networks underlying these outcomes [181].

Evidence from first-episode schizophrenia indicates that initiating clozapine after a first relapse significantly reduces the risk of subsequent relapse compared with continuing or switching non-clozapine oral antipsychotics [182]. Similarly, long-acting injectable antipsychotics have demonstrated superiority over oral formulations in reducing relapse and hospitalization rates, even among early-phase patients [183]. Additionally, meta-analytic data suggest that risperidone is particularly effective in first-episode schizophrenia, showing higher response and remission rates compared with control groups, though at the cost of increased risk for extrapyramidal symptoms and weight gain [184]. Collectively, these findings emphasize the importance of early, personalized treatment selection, balancing efficacy and tolerability, to optimize adherence, prevent relapse, and support long-term functional recovery.

Recent advances in neuroscience, genomics, and digital health are driving a shift toward integrative and precision-oriented models of schizophrenia. By combining multimodal data, including neuroimaging, genetic, immune, and behavioral markers, researchers aim to better capture the disorder’s biological and clinical heterogeneity [185,186].

Artificial intelligence and machine learning play a central role in this process, enabling the integration of large, complex datasets to identify predictive biomarkers and individualized treatment profiles [187,188]. Explainable AI approaches further improve interpretability, allowing clinicians to translate computational insights into practical decision-making.

This integrative framework supports a transition from static diagnosis to continuous, data-driven mental health management. Future directions should emphasize the creation of interoperable, ethically guided systems that link biological mechanisms, digital monitoring, and personalized interventions [189].

## 3. Discussion

This article synthesizes contemporary evidence on the neurobiological, clinical, and therapeutic dimensions of schizophrenia, highlighting the shift from a dopamine-centric disorder toward a multidimensional neuropsychiatric syndrome involving distributed neurotransmitter systems, circuit-level dysfunction, and complex gene–environment interactions. The convergence of molecular, neuroimaging, and clinical findings underscores that schizophrenia cannot be adequately explained by a single pathogenic pathway but rather reflects the cumulative impact of dysregulated neurodevelopmental and neurochemical processes.

A central theme emerging from the literature is the pivotal role of disrupted excitation–inhibition balance, particularly involving GABAergic interneurons and their regulation of cortical network oscillations. Reductions in GAD67 expression, impaired parvalbumin interneuron function, and altered gamma oscillatory activity collectively provide a mechanistic substrate for cognitive deficits and working memory impairment. These findings reinforce the concept that schizophrenia involves a failure of coordinated cortical information processing rather than isolated neurotransmitter abnormalities. Importantly, the observed interactions between GABAergic dysfunction, glutamatergic dysregulation, and dopaminergic signaling help reconcile long-standing discrepancies between classical dopamine models and more recent circuit-level theories.

Beyond neurotransmission, growing evidence implicates immune dysregulation, oxidative stress, and altered neurodevelopmental trajectories as core contributors to disease vulnerability. Microglial dysfunction, inflammatory signaling, and disrupted synaptic pruning appear to intersect with genetic susceptibility, reinforcing the view of schizophrenia as a neurodevelopmental disorder with progressive features. These biological processes also provide plausible mechanistic links to emerging biomarkers, such as altered iron metabolism, immune signatures, and neuroimaging markers of cortical and subcortical dysfunction.

From a therapeutic perspective, the reviewed evidence underscores both the strengths and limitations of current pharmacological strategies. While antipsychotics remain indispensable for controlling positive symptoms, their limited efficacy in addressing negative and cognitive symptoms highlights an urgent need for novel targets. Advances in glutamatergic modulation, muscarinic receptor agonism, and trace amine-associated receptor (TAAR1) agonists represent promising directions that move beyond dopamine antagonism. Equally important is the growing recognition that pharmacotherapy alone is insufficient; integrated psychosocial interventions, including cognitive remediation, supported employment, and family-based therapies, are essential components of effective, recovery-oriented care.

The increasing emphasis on precision psychiatry marks a paradigm shift in schizophrenia research and treatment. The integration of genomic data, neuroimaging biomarkers, digital phenotyping, and machine-learning-based predictive models offers the potential to stratify patients according to biological and clinical profiles, enabling more personalized interventions. However, translating these advances into routine clinical practice requires careful validation, ethical oversight, and equitable access to emerging technologies.

Contemporary evidence positions schizophrenia as a complex, heterogeneous disorder arising from dynamic interactions among neurodevelopmental, neurochemical, and environmental factors. Progress in understanding its pathophysiology has been substantial, yet meaningful clinical transformation will depend on bridging mechanistic insights with individualized, recovery-oriented care. Future research must prioritize integrative models that combine biological, psychological, and social dimensions, ensuring that advances in neuroscience translate into tangible improvements in long-term outcomes and quality of life for individuals living with schizophrenia.

## 4. Materials and Methods

This review was conducted using a PRISMA-informed narrative synthesis framework, adapted to accommodate the conceptual breadth and heterogeneity of schizophrenia research. The approach allowed structured reporting of the search strategy while retaining flexibility for thematic synthesis across distinct chapters.

A structured literature search was conducted in PubMed during December 2025, covering publications up to December 2025. Searches were performed using thematic Boolean combinations, with the full-text filter applied from the outset and restricted to English-language publications.

Representative search strings included: ‘schizophrenia’ AND ‘dopaminergic dysfunction’ (3660 results), ‘schizophrenia’ AND ‘GABAergic dysfunction’ (698 results), ‘schizophrenia’ AND ‘serotonergic dysfunction’ (329 results), ‘schizophrenia’ AND ‘glutamatergic dysregulation’ (223 results), ‘schizophrenia’ AND ‘antipsychotic treatment’ AND ‘first-episode’ (31,775 results), ‘schizophrenia’ AND ‘biological subtypes’ (792 results), ‘schizophrenia’ AND ‘digital health’ (684 results). Additional targeted searches were conducted using broader conceptual terms (e.g., treatment guidelines).

Across all searches, 38,161 records were identified. After title and abstract screening, 37,783 records were excluded, resulting in 378 articles eligible for qualitative synthesis. Following relevance-based refinement and chapter-specific selection, 155 sources were retained for inclusion in the final narrative synthesis, comprising reviews, and clinical practice guidelines incorporated a priori for the treatment section. Study selection followed a two-stage screening process (title/abstract followed by selective full-text assessment), informed by PRISMA principles but adapted to a chapter-specific inclusion strategy. Neurobiological mechanism sections prioritized: high-impact narrative and systematic reviews synthesizing convergent evidence, human neuroimaging and post-mortem studies and translational animal models with clear mechanistic relevance. Selection emphasized biological plausibility, methodological rigor, and theoretical integration, rather than exhaustive inclusion. Treatment and guideline section focused on: official international clinical guidelines (APA, NICE, CINP, WFSBP, McCutcheon et al.), large randomized controlled trials, meta-analyses with direct clinical applicability. Priority was given to recency, clinical relevance, and methodological transparency. Clinical practice guidelines (APA, NICE, CINP, WFSBP), consensus documents, major meta-analyses, and standard psychopharmacology reference texts (e.g., Stahl) were included a priori for the treatment chapter, independent of the structured database search, given their normative role in clinical decision-making. Emerging and future-oriented sections included: precision psychiatry, biomarkers, AI, and digital phenotyping, early translational or proof-of-concept studies and authoritative expert reviews.

The inclusion criteria were: peer-reviewed original research articles, systematic reviews, and meta-analyses; studies addressing the neurobiological mechanisms (dopamine, GABA, serotonin, etc.) of schizophrenia, clinical trials and reviews on pharmacological treatments, including first-generation, second-generation, and emerging non-D_2_ agents, studies on non-pharmacological interventions (CBTp, CRT, family interventions, IPS); comparative reviews or original guideline publications from major international bodies: APA (2024), NICE (2023), CINP (2023), WFSBP (2019), and McCutcheon et al. (2025).

Exclusion criteria: case reports, editorials, letters, or conference abstracts lacking full methodology; studies focused exclusively on non-schizophrenia psychotic disorders unless directly relevant to shared neurobiological mechanisms; non-English language publications that did not offer a reliable English summary.

Findings were synthesized thematically and compared across chapters, allowing both well-established evidence and emerging concepts to be integrated across neurobiological, clinical, and prognostic domains.

## 5. Conclusions

This review aimed to integrate recent evidence on the neurobiological mechanisms of schizophrenia, with a focus on dopaminergic circuit dysfunction, symptom heterogeneity, and treatment implications. The findings synthesized from molecular imaging, neuroimaging consortia, and longitudinal clinical studies strongly support the view that schizophrenia is characterized by regionally specific and developmentally dynamic disturbances in dopamine signaling, rather than a global dopaminergic abnormality.

Consistent with the results reviewed, presynaptic dopaminergic dysfunction—particularly elevated dopamine synthesis and release capacity within striatal regions—has emerged as one of the most robust and replicated neurobiological findings in schizophrenia. Meta-analytic and imaging evidence indicates that this abnormality is closely linked to positive psychotic symptoms, while dopaminergic alterations outside the striatum, including reduced cortical dopaminergic signaling, are implicated in negative and cognitive symptom domains [6,20]. These findings refine classical dopamine hypotheses and help explain why current antipsychotic treatments are effective for positive symptoms yet largely ineffective for cognitive impairment and motivational deficits.

Results from large-scale neuroimaging studies further demonstrate that schizophrenia involves widespread cortical and subcortical abnormalities, particularly within frontostriatal and frontoparietal networks that support executive and cognitive functioning. Data from international consortia confirm reproducible structural and functional alterations in prefrontal and temporal cortices, reinforcing circuit-based models of disease pathophysiology [174]. However, despite consistent group-level findings, the reviewed literature highlights a persistent translational gap: neurobiological markers have not yet achieved sufficient precision to guide individualized treatment selection or reliably predict clinical outcomes.

Clinical outcome studies reviewed here underscore the significance of treatment timing. Evidence from recent longitudinal cohorts indicates that delayed initiation of clozapine in treatment-resistant schizophrenia is associated with poorer long-term outcomes, including higher rates of psychiatric rehospitalization. Conversely, earlier clozapine initiation is linked to improved clinical stability, supporting calls for earlier recognition of treatment resistance and more timely use of clozapine [94]. In parallel, large observational studies and registry-based analyses consistently demonstrate that clozapine treatment is associated with lower all-cause mortality and reduced suicide risk compared with other antipsychotic medications, reinforcing its unique role in the management of severe illness [95,190].

As a result, the findings reviewed support a reconceptualization of schizophrenia as a disorder of distributed neural circuit dysfunction with distinct neurochemical and clinical stages. While advances in dopaminergic and systems-level neuroscience have substantially refined pathophysiological models, major unmet needs remain—particularly in the treatment of negative symptoms, cognitive dysfunction, and functional recovery. Future progress will require longitudinal, multimodal approaches that integrate clinical phenotyping with neuroimaging, molecular, and real-world outcome data. Bridging the gap between mechanistic insight and clinical implementation—especially through earlier, evidence-based interventions—represents a critical priority for improving long-term outcomes in schizophrenia.

## Figures and Tables

**Table 1:** Clinical Characteristics and Adverse Effects of Atypical Antipsychotics.

Agent	Clinical Profile	Notable Adverse Effects
Risperidone/Paliperidone	Potent D_2_, 5-HT_2_A blockade; good for aggression [72,73]	Hyperprolactinemia, EPS [72,73]
Olanzapine	Broad receptor binding; superior efficacy [72,74]	Weight gain, metabolic syndrome [72,74]
Quetiapine	Sedating, mood-stabilizing [72,75]	Orthostatic hypotension, sedation [72,75]
Ziprasidone	Pro-cognitive, low metabolic risk [72,76]	QTc prolongation [72,76]
Lurasidone	Pro-cognitive, minimal weight gain [74,77]	Akathisia, nausea [74,77]
Aripiprazole/Brexiprazole/Cariprazine	D_2_ partial agonists, serotonin modulation [73,78]	Akathisia (aripiprazole), insomnia [73,78]
Asenapine	Sublingual; good for mixed symptoms [73,79]	Oral hypoesthesia, taste alteration [73,79]

Abbreviations: EPS = extrapyramidal symptoms; QTc = corrected QT interval; D_2_ = dopamine D2 receptor; 5-HT_2_A = serotonin 5-HT2A receptor.

**Table 2:** Comparison of schizophrenia treatment recommendations across major guidelines.

Dimension	APA (2024) [63]	NICE (2023) [55]	CINP (2023) [136]	WFSBP (2019) [56]	McCutcheon (2025) [61]
Pharmacologic Focus	SGAs, early clozapine in TRS	Shared decision, both FGAs/SGAs	Receptor-targeted SGA choice	Hierarchical model	Precision, biomarker-based
Psychological Interventions	CBTp, psychoeducation	CBTp (≥16 sessions)	CBTp, CRT	CBTp, stress management	AI-augmented CBT
Family Involvement	Psychoeducation, relapse prevention	10+ structured sessions	Psychoeducation	Family support	Digital family support
Digital Integration	Telepsychiatry, monitoring	Activity tracking	Blended AI approaches	Limited	Digital phenotyping
Recovery Orientation	Shared decision, supported employment	Co-production, arts therapy	Functionality-focused	Community engagement	Holistic, precision recovery

Abbreviations: SGA = second-generation antipsychotic; FGA = first-generation antipsychotic; TRS = treatment-resistant schizophrenia; CBTp = cognitive-behavioral therapy for psychosis; CRT = cognitive remediation therapy.

## Data Availability

No new data were created or analyzed in this study. Data sharing is not applicable to this article.
